# Activity Recognition in Residential Spaces with Internet of Things Devices and Thermal Imaging

**DOI:** 10.3390/s21030988

**Published:** 2021-02-02

**Authors:** Kshirasagar Naik, Tejas Pandit, Nitin Naik, Parth Shah

**Affiliations:** 1 Department of Electrical and Computer Engineering, University of Waterloo, Waterloo, ON N2L 3G1, Canada; tnpandit@uwaterloo.ca (T.P.); pp3shah@uwaterloo.ca (P.S.); 2 School of Informatics and Digital Engineering, Aston University, Birmingham B4 7ET, UK; n.naik1@aston.ac.uk

**Keywords:** thermal images, activity recognition, 3D scene reconstruction, 3D thermal model, Internet of Things (IoT)

## Abstract

In this paper, we design algorithms for indoor activity recognition and 3D thermal model generation using thermal images, RGB images, captured from external sensors, and the internet of things setup. Indoor activity recognition deals with two sub-problems: Human activity and household activity recognition. Household activity recognition includes the recognition of electrical appliances and their heat radiation with the help of thermal images. A FLIR ONE PRO camera is used to capture RGB-thermal image pairs for a scene. Duration and pattern of activities are also determined using an iterative algorithm, to explore kitchen safety situations. For more accurate monitoring of hazardous events such as stove gas leakage, a 3D reconstruction approach is proposed to determine the temperature of all points in the 3D space of a scene. The 3D thermal model is obtained using the stereo RGB and thermal images for a particular scene. Accurate results are observed for activity detection, and a significant improvement in the temperature estimation is recorded in the 3D thermal model compared to the 2D thermal image. Results from this research can find applications in home automation, heat automation in smart homes, and energy management in residential spaces.

## 1. Introduction

Activity recognition and 3D thermal model generation are two important applications for which thermal images can be used. Activity recognition aims to detect the movement of an object or human in a sequence of images or video. It can be applied to various fields such as security, surveillance, sports, healthcare and household monitoring, etc. [1]. Activity recognition in the indoor environment such as home, office involves the detection of human activity and heat activity around household appliances. Human activity can be recognized using various technologies namely sensors, video cameras, etc. [2], and using traditional or machine learning approaches [3,4]. Human activity recognition (HAR) is an important module for home monitoring systems for smart homes. These types of systems can be used to provide safety to children and elderly people living in the home [5]. The combination of two emerging fields, Internet of Things (IoT) and Machine learning has achieved promising results for HAR [4,6]. The main data source for the task of activity recognition is sensors. There are two types of sensors used for this purpose: one is external and the second is wearable [7]. Wearable sensors are used in various healthcare applications such as patient monitoring, fitness monitoring, oxygen level monitoring, heartbeat monitoring, etc. External sensors are fitted in a static place from where the region of interest can be monitored. These types of sensors include cameras, thermal infrared sensors, etc. This paper uses thermal and RGB camera as sensing devices to capture thermal and visual information of a scene and fitted to a vantage point.

In addition to HAR, for indoor environment, household activity detection plays an important role. Household appliances such as the oven, television (TV), and stove generates heat which warms the surrounding environment. By capturing the temperature fluctuations around these objects any thermal activity can be detected. Thermal infrared images capture temperatures using infrared sensors. These images can be used to extract temperature fluctuations information which can be used for activity detection and recognition. Heat activity recognition in the kitchen increases safety as there are many electrical appliances placed in the kitchen [5]. In addition to hazard detection, it can be used for energy conservation and for modeling energy performance as well [8]. Moreover, the duration of each activity can be calculated and can be used to detect any high-risk situation. For example, if a burner is on a high-temperature state for more than a certain duration it can cause a fire-related hazardous situation. Although thermal images are used to detect heat leakage, IoT devices are used to alert the user [9]. These applications of activity recognition using thermal images and the IoT devices, contribute to building smart and safe homes.

One limitation of detecting activity using 2D Thermal images is that it provides information for X-Y image coordinates only. For example, to detect heat or energy leakage around an electrical appliance, one more dimension of depth is needed. Modeling the 3D thermal model enables us to measure energy performance [8] and to perform volumetric heat measurement [10]. A 3D thermal model consists of 3D points, reconstructed from images of a scene, with the corresponding temperature value. It describes the heat distribution of a scene in 3D. The 3D thermal model provides temperature information about the depth of each object in a scene. To reconstruct a 3D scene, various techniques such as 3D laser scanning using omnidirectional vision [11], structured light technology [12], etc. are used. 3D laser scanning requires a laser scanner that would scan the whole scene and create its 3D model. Stereo images can be used to reconstruct a 3D scene using principles of photogrammetry.

The overall objective of the paper is to determine the activity in a scene and to find the heat distribution in 3D. The thermal images are used to identify the activities such as heat or energy leakage for the appliance in operation which cannot be detected by only inspecting the visuals. In the first phase, we find out all the objects which act as either heat sink or heat source that are present in a scene and generate a corresponding file containing the information about the objects such as their name, temperature, and location in the image. Then, activity state and its duration are detected by applying various algorithms on generated information. In the second phase, the paper proposes to create a 3D thermal model using 3D scene reconstruction and temperature extrapolation to extract depth temperature information of a scene. The 3D thermal model is generated from the RGB stereo and thermal images captured using FLIR ONE PRO camera. The 3D thermal model construction involves many steps and one of the crucial steps is to find intrinsic and extrinsic camera parameters for both thermal and RGB cameras. RGB camera parameters can be calculated using the checkerboard method proposed by [13]. However, the thermal camera requires a different setup for this purpose. Thus, we have used Python APIs of the Metashape tool [14] to construct 3D mesh and Meshlab [15] to generate 3D thermal model without manually calibrating the camera to find its parameters. The temperature around the heated object can be known to identify any hazardous condition by using this thermal cloud. Thus, by using the thermal and RGB images, and processing them in an IoT-based setup we can achieve our objective of activity recognition. Such data of detected activities can be used for home automation (HA) purposes such as to control stove temperature to reduce the risk of hazardous situation, to automate heating, ventilating and air conditioning (HVAC) for human well-being [16]. Human activity states and its duration can be used for energy saving tasks such as controlling lighting, televisions, and other electrical appliances [17].

This paper makes the following contributions:

1. A new approach for human and heat activity recognition by using sequential thermal-RGB image pairs of a scene.
2. Algorithms to recognize activity states with its duration to determine hazardous conditions due to heat leakage around electrical appliances in indoor environments.
3. A 3D thermal scene reconstruction approach to project temperature of a scene in 3D space from 2D thermal and RGB images to gain more insights about the heat leakage.

The main purpose of this study is to automate heat management in the smart home and office. The proposed algorithms’ output can be provided to other software or hardware for various applications in smart homes and offices. For an instance, the results of human activity recognition can be linked with the HVAC system to control the temperature of the room based on the person’s activity. If the person is sleeping or lying down, the temperature should be adjusted to increase comfort. Another instance is that the results of heat activity recognition can be provided to the alert systems to detect and prevent any hazardous situation. If a person is sleeping and a heat leakage is detected in any of the electrical appliances then the system can automatically alert the concerned authorities and can turn off the damaged electrical appliance to prevent major accidents.

The paper is further divided into four sections. The literature review section discusses all the existing methods for activity recognition and 3D thermal model generation. In the Materials and Methods section, the overall flow of the experiment and algorithms used in this paper is provided and discussed. The Results and Discussion section contains description of experiments and discussion on results. Lastly, the challenges section describes all the challenges faced and their possible solutions.

## 2. Literature Review

In recent years, systems have been developed to recognize human and stove activity in smart homes. This type of system increases the safety of the home [5,18,19]. Some of these proposed systems have used RGB images [20] and some have used thermal infrared images [21,22,23,24]. In particular, thermal images were found to be useful to detect activity around appliances as they generate heat that can be easily detected in thermal images [5,19].

In [19], thermal images were used to detect activities of appliances such as a stove, refrigerator, oven, etc. Each appliance in a room was assigned a region of pixels in a thermal image. The temperature change in a region signifies the activity on the appliance. The system performed well but it was not designed to detect any human activity. In [25], a simple array of infrared sensors was used to detect moving, sitting, and standing activity of a person. The use of these sensors to generate thermal images solves privacy concerns and provide accurate results. Reference [5] proposed the design of an alert system to reduce fire hazards in homes especially for elderly people. This system has a FLIR camera fitted such that it can capture thermal images of the stove-top. The whole system is divided into sub-systems each performing operation on input thermal images to recognize different activities around the stove. For example, if the stove was left active for a specific amount of time then the system will detect it and send an alert message to the user. The results suggest that thermal images give better performance than RGB images to detect stove activity as the thermal image is capable of capturing temperature changes whereas RGB images failed for this objective. However, the overall alert system depends on the output of multiple sub-systems. Thus, the system is not robust. To detect the presence of an animal, thermal images were used in [26]. Similarly, it can be used to detect pet animals in a house. Apart from this, thermal images have applications in the field of activity recognition such as heat transfer through building walls [27], detection of gas leakage in industries [28], and in surveillance and security [29].

Many studies have used different approaches for feature extraction from a video to recognize the activity. References [22,23] proposed to use Gain Energy Image (GEI) which was created from a sequence of images. Furthermore, reference [22] has used Principal Component Analysis (PCA) and Multiple Discriminant Analysis (MDA) to extract and reduce dimensions of a GEI, resulted in improved performance. Statistical methods were used to extract features in [24] to detect human activities such as Stand, Sit, Walk, Bend, Run, Raise hands, and Kick. However, very few studies have used a Convolutional Neural Network (CNN)-based approach to extract features in thermal infrared images. CNNs can extract complex features from the visible image. However, in thermal images, it is difficult to train CNN to extract complex features as a thermal image does not contain features such as edges or corners. However, statistical approaches mentioned in [24] does not suffer from this problem. However, many papers designed new methods to solve these problems of thermal images and used combination of CNN and Recurrent Neural Network (RNN) for HAR [30,31].

After extracting the features from the thermal image or video some researchers have used model-based and others have used non-model-based approaches. Reference [20] compared different machine learning (ML) algorithms for Human Activity Recognition (HAR). Multilayer Perceptron or Artificial Neural Network performed best among all other algorithms. This paper proposed to use changes in human postures during an activity as features for ML algorithms. RGB images were used instead of the thermal images which resulted in reduced performance as the color of the background and foreground can affect human-body detection. Reference [21] proposed to use thermal images for HAR in darkness. OpenPose library was used to detect 18 different joints of the human body. Features such as angles between two joints, change in those angles during different time frames was calculated and fed into a deep neural network as features. The method performed well with dark background, but the camera needs to be calibrated before recording a video as an image covering a full human body is required to correctly recognize the human activity. Results suggested that RNN performed best among all other deep learning algorithms. There are many ways to complete the task of HAR. Reference [18] gives a review and comparison of different approaches.

Table 1 shows that to achieve the goal of human and household activity recognition different papers have considered different techniques. In the table, the first three rows classify papers by the input data used. References shown in the first row used thermal images, in the second row used RGB images and in the third row used infrared sensor data. The next three rows categorize papers by the method used for activity recognition. Fourth, fifth and sixth rows describe references that have used deep learning, machine learning, and custom algorithms for activity recognition, respectively. The last row of the table represents the papers in which the authors have determined the activity duration for human and household activity to achieve their objectives. The two columns indicate the type of activity recognition considered in the papers. One is human activity and the other is household activity recognition. It can be depicted from Table 1 that most of the papers proposed to use thermal imaging [21,22,23,24], and machine learning [18,20,22,23,24,25,32] for human activity recognition whereas for household activity recognition custom algorithms [5,26] were used. Custom algorithms use classical image processing methods to detect and recognize activities. References [5,19,32] have measured activity duration for each activity and used it in the algorithm to solve the problem.

Furthermore, reference [33] used Support Vector Machine (SVM) with Radial Basis Function (RBF) kernel to recognize human actions such as bend, jack, jump, run, side, skip, walk and wave. The paper used the Weizmann, Soccer, and Tower datasets, and gained 100% accuracy on the Weizmann dataset. The main objective of the paper is to recognize human actions from a far-field of view. Reference [34] proposed a new approach for human action recognition using histogram of oriented optical flow from the input video. The paper also used the Weizmann dataset for experimentation and to evaluate their approach. This paper recognized the same activities as reference [33] and achieved 94.4% accuracy on the Weizmann dataset. Reference [35] proposed an optical flow-based human activity recognition approach and achieved 95.69% and 94.62% accuracy on the Weizmann and KTH dataset, respectively. Reference [36] proposed to use histograms of the optical flow extracted from the input video. The paper used the K-nearest neighbor algorithm to classify human activity recognition using the features extracted from the motion vectors and achieved 79.17% accuracy on the Weizmann dataset. Reference [37] proposed to use backpropagation neural network using the features extracted by histograms of oriented gradients and achieved 99.17% accuracy on the Weizmann dataset. Reference [38] presented a novel approach for human action recognition using spatial-temporal features and experimented on the Weizmann dataset achieving 72.8% accuracy. Reference [39] proposed to use optical flow-based segmentation and achieved 90.32% accuracy on the Weizmann dataset. Human motion trajectories are used in the reference [40] to recognize human action. The authors experimented on four datasets namely Hollywood2, HMDB51, Olympic Sports, and UCF50, and achieved an accuracy of 64.3%, 57.2%, 91.1%, and 91.2% respectively. However, our approach focuses on indoor human activities, heat activities, and 3D model reconstruction. To validate our approach, we created an experimental setup and created our own dataset to validate the human activity recognition algorithm.

For the application of HAR, many researchers have used data generated from wearable sensors [41]. Sensors generate a large amount of data in each time step t, creating a requirement to study efficient algorithms for data analysis [41]. To detect physical activity for healthcare purposes, reference [42] proposed to use gyroscope and accelerometer sensors. These sensors help to examine and understand daily routine life and different postures. However, data generated through these sensors need to be cleaned and transformed. For the task of activity recognition, feature extraction from these sensor data is done using traditional statistical methods in [42]. Reference [43] used accelerometer-based sensors attached to the human body to detect various activities for patient monitoring in hospital environments. The sensor was attached to the human trunk and used to measure different body movements of the patient. Reference [43] proposed to recognize activities such as body posture, whether the patient is on movement or not, the patient is using a wheelchair or not, etc. The main objective of the paper was to apply different machine learning algorithms and compare their results to find the best performing algorithm on sensor-generated data. With the increasing popularity of smartphones, the research in the field of smartphone sensor data analysis has found many applications. Reference [44] used smartphone sensor data placed in the pocket and hand to recognize locomotion-based human activities. The study used various sensors such as accelerometer and gyroscope to gather and analyze human motion and posture data. One advantage of using these sensors is that it can work in any indoor and outdoor environment. However, these studies are mainly focused on human activity recognition and are not aimed to recognize household activities.

Reference [45] used RGB-D sensor to get depth information and to detect human activity from the gathered data. The paper proposed to recognize multiple activities using only one sensor device called Microsoft Kinect for Windows V2. However, the paper does not aim to track the activity of each person and to detect heat activity in the indoor environment. Also, the application of RGB-D sensors in a darker environment significantly affects accuracy. Reference [46] proposed to use a 32 × 32 thermopile array to detect human activity in indoor environments. The sensor array is fitted to a wall such that the scene can be observed, and the sensor can detect only activities happening in the 33°× 33° field of view [46]. The sensor can perform well in darkness as it detects infrared waves. However, this array of sensors monitors a very small region of a room and does not provide any visual information about household appliances. To reduce the size of the input data and to improve computational efficiency, reference [47] used pyroelectric infrared sensor array and converted the sensed data into binary. This binary data is then given to the convolutional neural network to extract important features and to recognize human activity. The paper achieved very good accuracy, but it does not contain information about objects of a scene and is not aimed to recognize the number of persons present in the room. Due to increased availability and high-speed internet, various research has studied the method of remote monitoring of the daily activity of a person. Reference [48] used thermal sensors fitted in the kitchen and living room to monitor human activities throughout the day. The produced sensor data were sent to a remote location for further processing and analysis. Sensors can be used in various fields to gather and analyze every human activity. Reference [49] proposed and validated an IoT-based approach to detect water consumption in a house for water conservation purposes. To achieve the task, the study used a water-flow sensor that gathers data and with the help of a microcontroller, the data is then sent to the internet so that the consumer can see their usage and can keep track of each activity with water consumption.In this study, we proposed to use both RGB and thermal cameras with infrared sensors to get both visual and temperature information of a scene and processed this information to recognize activity and heat leakage.

Some studies have shown that heat energy measurement can be done more accurately using 3D thermal models. Many papers have shown that RGB and thermal images can be used to create a 3D temperature distribution model that will provide more accurate results for heat measurements. In [8,50], the 3D thermal model of interior and exteriors of a building was constructed and used for energy measurement using optical and thermal images. However, some approaches take more computational time to reconstruct a 3D temporal model compared to others. Many papers proposed new approaches to improve the performance for 3D thermal model reconstruction in terms of computational time as well as precision of the model. References [10,51] described two new approaches that improved the computational time for the generation of the 3D model but failed to improve the accuracy of the 3D model. All these approaches require manual calibration of the camera using the checkerboard method proposed by [13] to calculate parameters of the camera. To calibrate a thermal camera a special type of checkerboard needs to be designed as thermal images cannot capture edges and corners of the checkerboard. Some researchers proposed to create a checkerboard with lights at each corner and edges so that thermal cameras can easily be calibrated. However, reference [52] proposed a new method that would calculate camera parameters automatically. This method is robust.

The accuracy of the 3D model depends on extracting key-point features and then finding a pair of images that are then used to create a 3D model. Many papers have used the Scale-Invariant Feature Transform (SIFT) to detect features that are important and invariant to scale and rotation. A pair of images can be found by matching these key-features. However, this process creates many outlier points and false positives. Outlier points reduce the accuracy of the 3D model. To remove the outliers, reference [8] used Random Sample Consensus (RANSAC), reference [52] used M-estimator Sample Consensus (MSAC) which is a variation of RANSAC, reference [10] used epipolar geometry and epipolar constraints, reference [51] improved Belief propagation algorithm to optimize the outlier detection technique and reference [50] did not use any outlier detection technique which resulted in increased computational time as there are more points for 3D reconstruction. After getting a pair of images, a point in 3D is constructed using different methods. References [8,50,52] proposed to use structure from motion (SFM) to create a point in 3D space, whereas references [10,51] used concepts of epipolar geometry to calculate the coordinates in 3D space. Both approaches create a sparse or dense 3D model depending upon the number of images provided.

This paper deals with the problem of activity recognition by using object detection model; pre-trained on the COCO dataset, and our proposed custom algorithms. The pre-trained model is used on the captured images to extract information about the objects in a scene. Then, this information is used by our proposed algorithms to recognize activity states and their duration. For the task of 3D thermal model generation, the paper uses stereo RGB images and a thermal image. To capture thermal images the paper proposes to use the FLIR ONE PRO camera that has a thermal camera with thermal infrared sensors to capture thermal activities. The 3D model is generated by using tools such as Metashape and Meshlab. To extract temperature in 3D space, a custom algorithm is proposed.

## 3. Materials and Methods

**Figure 1:**
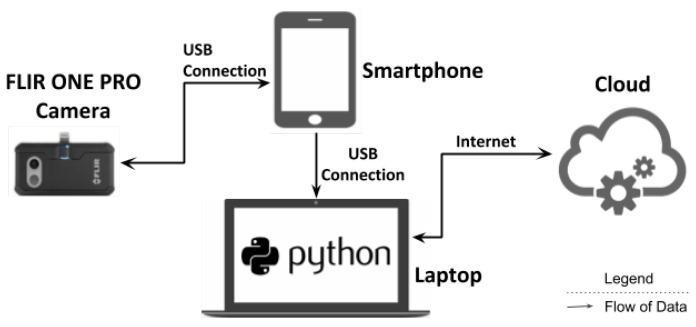
Experiment Setup.

This section describes framework deployment, interactions between different components and computations. The experimental setup for the paper is shown in Figure 1. We have divided experiments into two modules, first is for activity recognition and the second is for 3D thermal model generation. This paper proposed to use the FLIR ONE PRO camera as an external sensor to capture RGB and thermal images. The camera is mounted at an elevated location from which the scene can be captured. The camera is directly connected to the smartphone via USB. Here, the smartphone acts as a remote and monitor for the camera. Smartphone sends a capture signal to the camera to capture the image at timestamp *t* and then receives the image to be monitored on screen.

**Figure 2:**
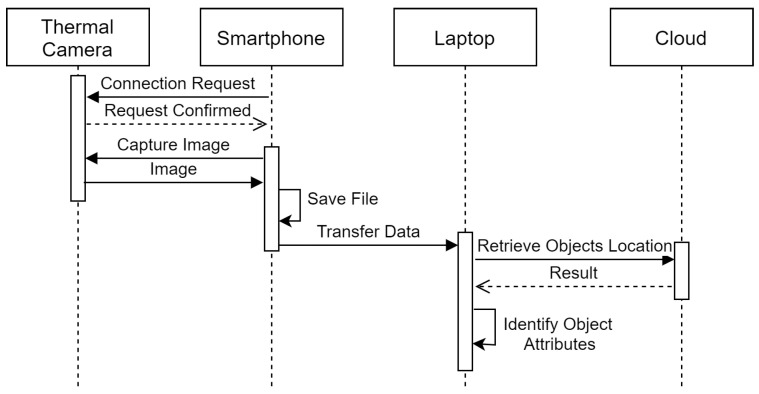
Interactions among the entities.

Figure 2 shows the use of devices in an IoT-based setup for first module. After capturing the scene the camera stores the raw images on the smartphone. Now, the images are transferred to the laptop via USB connection for processing. Processing is also divided into two parts. In the first part, the RGB images are sent to the cloud where object detection is performed. The tensorflow’s object detection API calls from the cloud return the bounding box and object name for the detected objects. This information along with both RGB and thermal image are given to the algorithms running locally on the laptop. Algorithms 1 and 2 produces the scene information file containing detected objects, their temperature, and location in an image.

**Algorithm 1** Generate Image Annotation

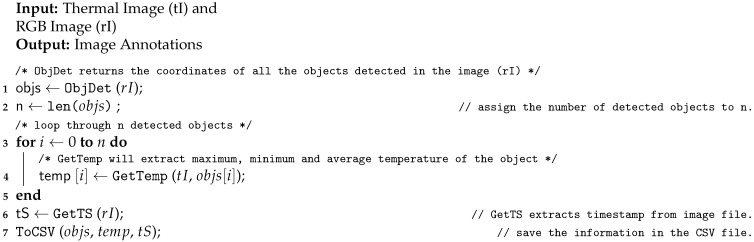



**Algorithm 2** Generate Video Annotation

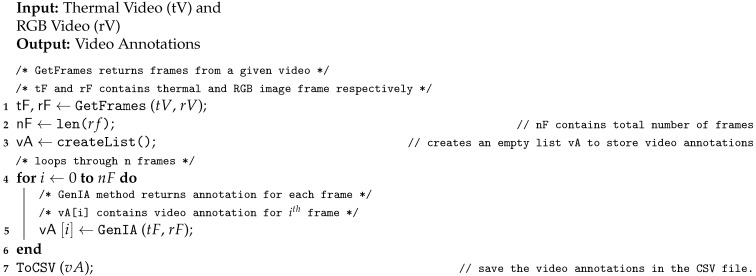



**Figure 3:**
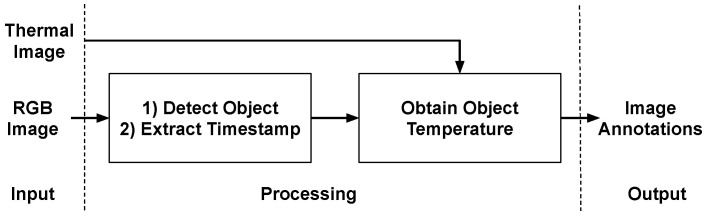
Workflow diagram for Image Annotations.

Computations and workflow of the experiment method for the first module are illustrated in Figure 3. Thermal and RGB images are passed on as input for the processing. Now, in the first part, object detection is performed on the RGB images. We have used tensorflow’s Object Detection API which is trained on COCO dataset. The COCO dataset contains 1000 different objects such as persons, bag-packs, smartphones, and tables. Given an RGB image as an input, the API gives the names and bounding box of the detected objects. We also extract the timestamp of the image as it will be important for activity recognition. At this point, we have the bounding box and object information for the scene. The only missing data is the temperature of that object which is obtained in the second part.

In the second part, the thermal image is used together with the output of the first part: the object detection information. Here, we iterate over all the detected objects and, using their bounding box (location in the RGB image), we obtain the object’s temperature by finding the max temperature value that the thermal image has in the bounding box. In this step, we assume that the RGB and thermal images are aligned. If the two are not aligned, then we must do image alignment before doing this process to make sure that the object location in RGB and the thermal image is the same. In the end, the temperature value of the object is added to the information file and image annotations are produced. Basically, Image annotations are the metadata about the image. Here, we are concerned with the details of the objects located in the image. For the task of activity recognition, we have extracted details such as the location (X-Y coordinates), height and width, minimum, maximum and average temperature of the object, and the timestamp of the frame. Thus, the objective to generate a file that contains all information (such as objects, locations, temperatures, and timestamps) from thermal and RGB image is achieved.

**Figure 4:**
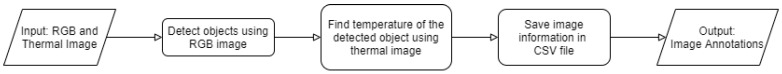
Flow diagram for Algorithm 1 (Generating Image Annotation).

Algorithm 1 describes the procedure for generating the image annotations that contain information about the detected objects and their attributes. Figure 4 depicts the flow of Algorithm 1. In Algorithm 1 the thermal image and the RGB image are given as input and it generates a CSV (Comma Separated Values) file containing image annotations. First, the RGB image is passed to the object detection function, which identifies the objects present in the image and their bounding boxes i.e., location in the image (line 1 in Algorithm 1). Now, for all the detected objects, their temperature values are obtained from the corresponding thermal image (block 3). A timestamp for the given image is obtained by inspecting the properties of the RGB image file (line 6). Then, all the information such as objects, bounding boxes, temperature values, and timestamps are saved as a CSV file (line 7). The saved CSV file contains tuples of the format (Object Name, X-coordinate, Y-coordinate, Width of the bounding box, Height of the bounding box, Timestamp, Maximum temperature, Minimum temperature, Mean temperature, Frame no).

**Figure 5:**
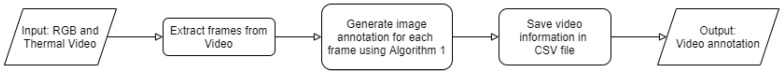
Flow diagram for Algorithm 2 (Generating Video Annotation).

We proposed to use video to detect an activity as it has temporal information that is useful for the task. However, Algorithm 1 requires input as an image to detect objects and generate annotations. Thus, we require the processing of the input video to extract frames. For the video processing, the thermal video and RGB video is given as an input to Algorithm 2 and it generates a CSV file containing video annotations. Figure 5 depicts the flow of Algorithm 2. First, RGB video and thermal video is processed to extract the respective frames (line 1 in Algorithm 2). Now, for all pairs of thermal and RGB frames, Algorithm 1 is used to annotate the frame (block 4). The generated video annotations are stored in a CSV file (line 7). The format of the tuples for this CSV file is the same as mentioned above for Algorithm 1.

**Figure 6:**
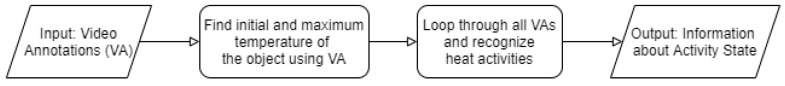
Flow diagram for Algorithm 3 (Heat Activity Recognition).

An activity can be defined as the change of state of an object with the change of time. It can be detected by recognizing state or temperature fluctuations in each frame. Algorithm 3 outlines the steps required to obtain the level of stove activity in each frame. Figure 6 depicts the flow of Algorithm 3. A stove can be in pre-heating state initially, steady state when temperature is steady, heating state when temperature rising, maximum temperature state, and cooling state while the temperature is decreasing. The workflow of the algorithm is simple: Initial frame’s temperature value is described as a pre-heating state (block 7 in Algorithm 3) and if it stays the same in the next few frames then it is described as a steady state (block 9). Then, when the temperature value rises from it but remains less than the max temperature then it is described as a heating state (block 12). Afterward, when the temperature values reach maximum it is labeled as a max temperature state (block 14). Lastly, when the temperature value starts to drop every frame, then it is described as a cooling state (block 16). The information regarding frame number and each state is stored in a CSV file (line 20) that has tuples in the form of (Object name, X-coordinate, Y-coordinate, Width of the bounding box, Height of the bounding box, Timestamp, Maximum temperature, Minimum temperature, Mean temperature, Frame number, Activity state). In the result section, we have shown only two columns: frame number and activity state for this CSV file.

**Algorithm 3** Heat Activity Recognition

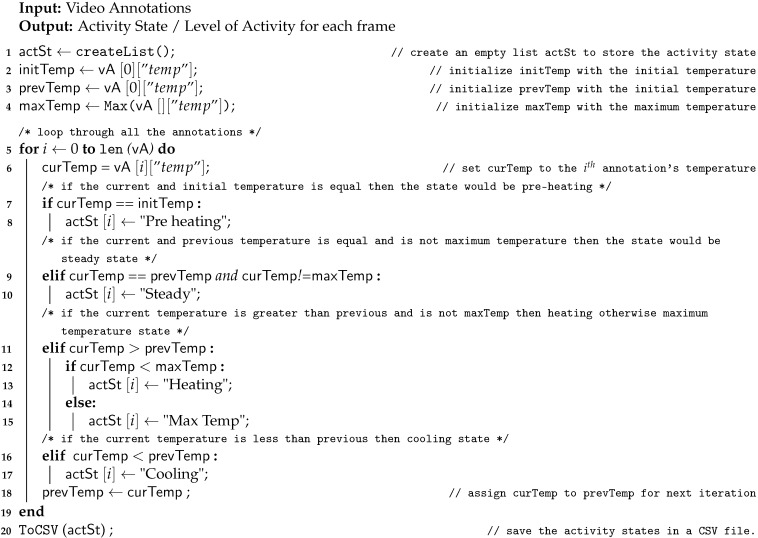



**Figure 7:**
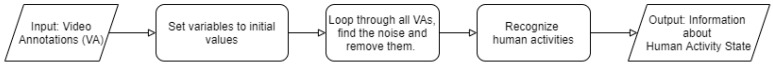
Flow diagram for Algorithm 4 (Human Activity Recognition).

We have implemented Algorithm 4 to recognize human activity given video annotations. Figure 7 depicts the flow of Algorithm 4. The algorithm detects how many persons are present in a room and recognize how many of them are sleeping, standing, sitting/resting. The flow of Algorithm 4 is simple. First, we group all the frames with a person in them by frame number (line 5 in Algorithm 4). Now, it is possible that the person in television and a photo frame is detected by the object detection module. We call this a noise and removed it (Line 7). After that, the total number of persons present in the frame were determined (line 8). Then, line 9 first checks whether a person is present in the current frame or not. If the person is present in the previous frame then block 9 will be executed otherwise block 27 will be executed. Block 11 checks whether a new person entered the current frame or not and counts the number of people. Block 12 checks if a person went away or not and counts the number of people who went out of the frame. Then, the algorithm will check for the states such as sleeping, standing, and resting. If the width of the bounding box is greater than the height of the bounding box after multiplying it by 1.7, then it can be said that the person is sleeping (Line 16). Here, our objective is to control the heat in the environment, so our algorithm is designed such that if the person is lying down or sleeping, we consider it as one category. If the height of the bounding box is greater than the doubled width then the person is standing. Otherwise, the person is resting or sitting. Line 29 detects and counts the number of people who left and line 31 detects that there is no person in the frame. Line 35 saves the activity state and frame number in a CSV file with annotation data. The format of the CSV file is the same as heat activity recognition.

**Figure 8:**
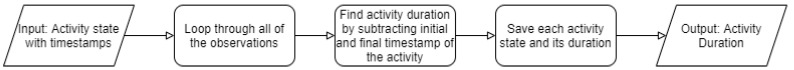
Flow diagram for Algorithm 5 (Activity Duration).

As mentioned in the introduction, activity duration can be used in applications such as kitchen and home safety [5,19]. We have implemented an Algorithm 5 to measure the duration of each activity. Figure 8 depicts the flow of Algorithm 5. This algorithm uses the CSV file created by Algorithm 2, Algorithm 3 or Algorithm 4, which contains each activity state and its timestamp. It first checks if the state is changed (line 8 in Algorithm 5) and if the state is changed then it will measure the duration for that activity by taking the difference between the first timestamp of the current activity and the first timestamp of the next activity (line 9). The algorithm is described in Algorithm 5. The output CSV file consists of tuples with two elements activity state and duration (line 14). The sample rows of the output CSV file generated by the algorithm are shown in the result section.

**Algorithm 4** Human Activity Recognition

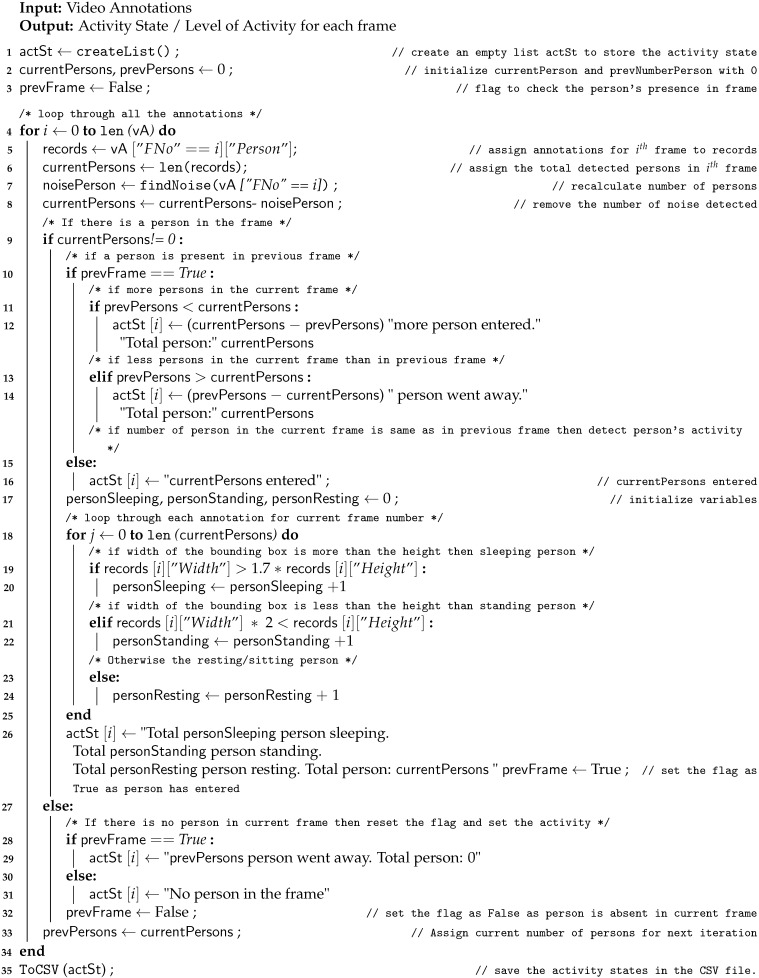



**Algorithm 5** Activity Duration

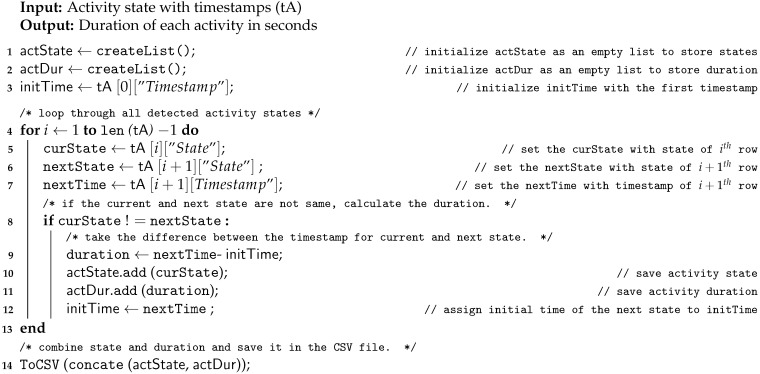



**Figure 9:**
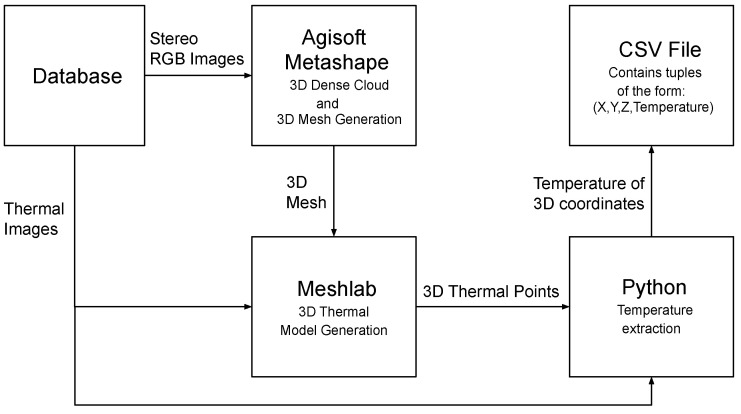
Flow Diagram for 3D thermal model.

The second module of the paper which deals with the 3D thermal model construction and 3D temperature extraction is divided into two steps. In the first step 3D scene is reconstructed in the form of 3D mesh from multiple stereo RGB images using Algorithm 6 and then the 3D thermal model is generated using 3D mesh and the thermal images. A 3D dense point cloud consists of 3D points of a scene and a 3D mesh represents the structure of the 3D scene. 3D mesh generation deals with the problem of surface generation given a 3D dense point cloud. The quality of 3D mesh depends on the quality of the 3D dense cloud. In the second step, the 3D thermal cloud and the 2D thermal image is given to the Algorithm 7 to extract the temperature of 3D coordinates based on its RGB hue and saves it in the form of CSV file with 3D coordinates and temperature. The flow for the process is shown in Figure 9.

**Figure 10:**
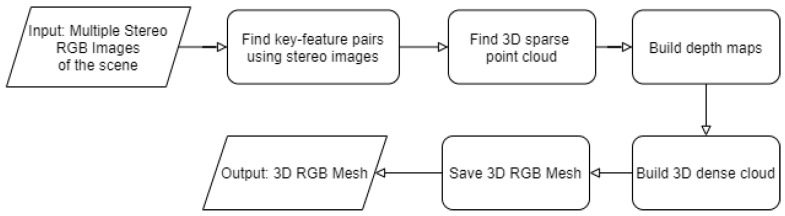
Flow diagram for Algorithm 6 (3D Mesh Generation).

In this paper, to generate a 3D point cloud and a 3D mesh, Python APIs of the tool named Metashape by Agisoft were used. The tool provides services to generate a 3D mesh. All the service calls are described in Algorithm 6. Figure 10 depicts the flow of Algorithm 6. Initially, all the stereo RGB images are loaded into the program to be processed (line 4 in Algorithm 6). The first step of 3D mesh generation includes finding the camera parameters and finding the camera location in the world coordinates. There are two camera parameters: intrinsic and extrinsic. These parameters map the 3D world coordinates to 2D image coordinate. Camera’s focal length, skew, lens distortion, and the center image coordinates are the intrinsic parameters [8]. Here, the assumption is that the images captured by the camera are not skewed and distorted. Thus, the value of the skew and the distortion parameters are assumed to be 0. Generally, these parameters can be written in the form of a 3 × 3 matrix which is shown below:

(1) $$K=\left[\begin{array}{ccc} f_{x} & 0 & c_{x}\\ 0 & f_{y} & c_{y}\\ 0 & 0 & 1 \end{array}\right]$$

where $f_{x}$ and $f_{y}$ are the focal length in pixel unit and $c_{x}$ and $c_{y}$ are the center image coordinates in pixel unit.

**Algorithm 6** 3D Mesh Generation **Input**: Multiple stereo RGB images of the scene **Output**: 3D mesh of a scene1 RGBImages ← createList();
//
 create an empty list to store image names
2 doc ← metashapeDocument();
//
 initialize metashape document
3 chunk ← metashapeChunk();
//
 initialize metashape chunk
4 chunk.addPhotos (RGBImages);
//
 add photos to the chunk object
5 chunk.matchPhotos();
//
 match key-features between pair of images
6 chunk.alignCameras();
//
 align cameras and generate sparse 3D point cloud
 /*
building depth maps using camera parameters and 3D sparse point cloud */7 chunk.buildDepthMaps();
8 chunk.buildDenseCloud();
//
 building 3D dense cloud using depth maps
9 chunk.buildModel();
//
 building 3D mesh using 3D dense cloud
 /* export 3D dense point cloud to the directory path provided by filePath */10 chunk.exportPoints (*filePath*);
 /* export 3D mesh to the directory path provided by modelPath */11 chunk.exportModel (*modelPath*);


Rotational and translational matrix are the extrinsic parameters of a camera [8]. Both the parameters can be written in the form of 3 × 3 and 3 × 1 matrix respectively which is shown below:

(2) $$R=\left[\begin{array}{ccc} r_{11} & r_{12} & r_{13}\\ r_{21} & r_{22} & r_{23}\\ r_{31} & r_{32} & r_{33} \end{array}\right]$$

where *R* is the rotational matrix that represents rotations in *X*, *Y* and *Z* coordinates.

(3) $$T=\left[\begin{array}{c} t_{1}\\ t_{2}\\ t_{3} \end{array}\right]$$

where *T* is the translational matrix that represents translation in *X*, *Y* and *Z* coordinates.

Using these three matrices, a 3D world coordinate can be mapped to the 2D image coordinate and vice-versa. The formula for the same is mentioned below:

(4) x=K[R|T]X

(5) $$\left[\begin{array}{c} x_{i}\\ y_{i}\\ 1 \end{array}\right]=K\left[R|T\right]\left[\begin{array}{c} X_{w}\\ Y_{w}\\ Z_{w}\\ 1 \end{array}\right]$$

where *x* is the vector of 2D image coordinates ($x_{i}$, $y_{i}$) and *X* is the vector with 3D world coordinates ($X_{w}$, $Y_{w}$, $Z_{w}$).

One of the tasks of 3D scene reconstruction is to find these intrinsic and extrinsic parameters that can re-project the 2D image coordinate to the 3D world coordinate system with minimal error. The Metashape tool follows the geocentric coordinate system for 3D models and in this system the origin (0, 0, 0) is located close to the projection centers [53].

The extrinsic parameters can be found by matching key-features from provided RGB images. Key-features are the features that are translational and rotational invariant. Thus, these features are most-common in all the captured images of the scene. These key-features were then used to find the rotational and translational matrix that is extrinsic parameters between every image pair. Line 5 describes this step using function call matchPhotos() that finds matching key-features. Line 6 describes the function call of aligning cameras that aligns every image, finds camera position in world coordinate, and generates a spare dense cloud using camera parameters. During the third step, a high-quality depth map was generated (line 7) and the fourth step builds a high-quality 3D dense point cloud using the depth maps generated in the previous step (Line 8 ). Here, high quality means that the images were not down-scaled to generate a more detailed 3D dense cloud. The last step generates the 3D mesh using the 3D point cloud generated in the previous step (Line 9). 3D dense point cloud and 3D mesh are saved at the directory path specified by filePath and modelPath, respectively.

Now, we have a 3D mesh with coordinates. However, these coordinates do not have an assigned hue. Hue is used to find temperature. A 3D model temperature assigned to its 3D coordinates is called the 3D thermal model. To create a 3D thermal model, the 2D thermal image is projected onto the 3D mesh created in the previous step. For this task, a tool named Meshlab is used. To project the thermal image, first, the texture from the thermal image is generated. Now, the patches of vertices from 3D mesh are re-projected to the texture and Meshlab will assign each vertex a color based on the re-projection. The re-projection might introduce some error as the 3D mesh is generated from the images of RGB camera whereas the re-projection is done using the images produced by the thermal camera. However, in FLIR ONE PRO, both the cameras are nearer to each other resulting in a small re-projection error.

The initial and the most important step to generate the texture is to align 3D mesh and 2D thermal image such that the center of the image and the center of the 3D mesh are aligned. This step requires camera parameters of the thermal camera. It can be achieved by designing a checkerboard with LED lights [8]. Another solution is to manually align both the coordinates. We have chosen the latter option and aligned them manually. After aligning them, the 2D image is projected onto the 3D mesh as mentioned above.

**Figure 11:**
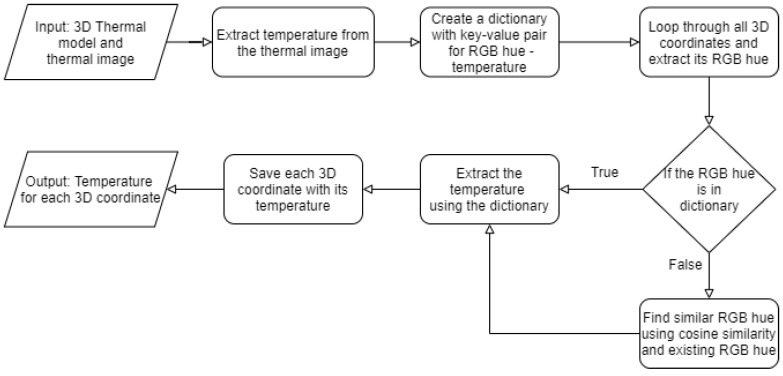
Flow diagram for Algorithm 7 (Temperature Extraction).

Now, we have a 3D thermal model from which we need to extract the temperature. To find the temperature from the 3D thermal model Algorithm 7 was designed. Figure 11 depicts the flow of Algorithm 7. The algorithm takes two inputs, one is a 3D thermal model and the other is 2D thermal images. The 3D thermal cloud contains 3D coordinates and RGB hue values. The algorithm first extracts temperature and its corresponding RGB hue value from the 2D thermal image (lines 2 and 3 in Algorithm 7) and reads the RGB hue of each 3D points from a 3D thermal model (line 1). Then, the algorithm maps RGB hue to its corresponding temperature from thermal image resulting in a dictionary with RGB value as a key and its corresponding value as the temperature (line 4). This dictionary is used to find the temperature of 3D coordinates (lines 10 and 13).

**Algorithm 7** Temperature Extraction

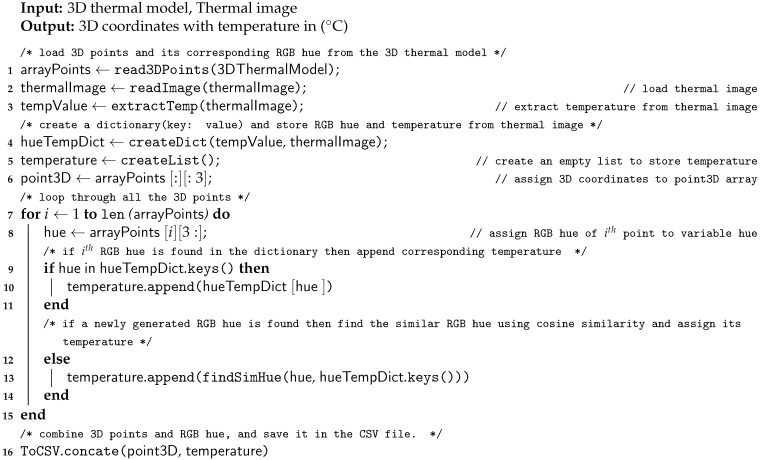



However, the 3D thermal model has some newly generated but similar RGB hue due to extrapolation. To deal with this problem similar RGB hue needs to be found from the extracted RGB hues of the thermal image. Reference [54] proposed a cosine similarity-based method to find similarity between two colors. Moreover, reference [55] used cosine similarity to find similar face colors. Thus, we proposed to use cosine similarity between the newly generated and existing RGB hues to find the best similar hue (line 13 in Algorithm 7). The equation for the same is shown below:

(6) $$\mathrm{Sim}\left(n,e\right)=\frac{n\cdot e}{∥n∥∥e∥}$$

Here, n is the vector containing the value of newly generated RGB hue and e is the vector containing the existing RGB hue value. Sim(n, e) is the value of cosine similarity between n and e. The similarity value is in the range of 0 to 1. 0 being the value for dissimilar colors and 1 being the value for most similar colors [54]. The temperature of the best similar hue was assigned to the temperature of the newly generated hue and to the corresponding 3D coordinate (line 13). This data is stored in the CSV file (line 16). The CSV file contains four columns, X, Y, Z coordinates, and one for temperature value in (°C) corresponding to the 3D coordinate. Results from all the algorithms are mentioned and discussed in the next section.

## 4. Results and Discussion

To validate the proposed algorithms, we have conducted a few experiments. The thermal images captured from the FLIR ONE PRO camera are in Multi-Spectral Dynamic Imaging (MSX) mode. This mode enhances the details of the thermal image by extracting high contrast edge details using the visual camera. The first experiment conducted was for heat activity recognition in the stove. An electric stove burner was left on and unattended. We have captured the video of this scene. This video is given as an input to Algorithm 2 that extracts the frame from the video and provide it as an input to Algorithm 1. Algorithm 1 detects objects in the input image and extracts information such as object coordinates, temperatures, and timestamps i.e., image annotation for each frame, and this information is returned to Algorithm 2 which combines all the annotations for all the frames into one output CSV file.

**Figure 12:**
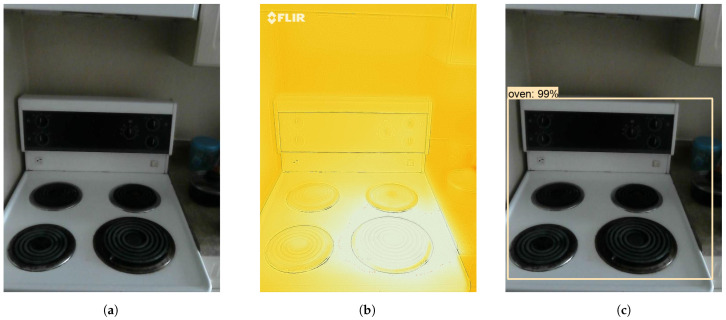
(**a**) Visual image, (**b**) Thermal image, and (**c**) Object detection of a stove/oven.

We have applied Algorithms 3 and 5 on the video annotations to find out when the burner was turned on (its temperature was climbing), when it reached its peak temperature and steady state, how long it was in its steady state, and when it was turned off. Now, this type of activity detection can be done only by using both thermal and visual information. This is because it is difficult to find out the temperature and state of the stove by using only visual information. Furthermore, it is difficult to tell due to which object the temperature is changing by using only thermal information. A sample frame for the stove experiment is shown in Figure 12a and its corresponding thermal image is shown in Figure 12b. Detected objects are shown in Figure 12c. The results of Algorithm 3 on stove experiment’s video annotations are shown in Table 2. The table depicts the activity state associated with a frame number. In frame-1, there is no activity, in frame-3 the stove started to heat up, in frame-12 stove reached the maximum temperature and in frame-18 the stove state is cooling i.e., the temperature starts decreasing.

**Figure 13:**
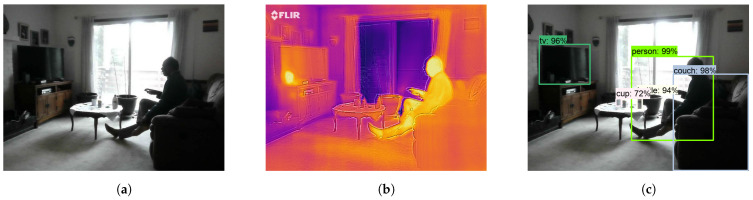
(**a**) Visual image, (**b**) Thermal image, and (**c**) Object detection in a living room.

We have also used video annotations to determine human activities using Algorithm 4 such as people entering or leaving a frame and standing or resting. A sample frame is shown in Figure 13a and its corresponding thermal image is shown in Figure 13b. Detected objects are shown in Figure 13c. The detected activity states of the human experiment’s video annotations are shown in Table 3. The table has two columns one is the frame number and the other is the activity state in that frame. For example, in frame-2, there is no human presence in the room and in frame-3 one person entered the room. For frame-4 one person is resting, in frame-26 one more person entered, in frame-59 one person is standing and one is resting, in frame-60 one person went away, in frame-148 one person is sleeping, in frame-153 one person is standing, and in the frame-232 the person went away. The activity state of frame-233 depicts that there is no person present. More results for video annotations of living room and kitchen are mentioned in Table A1 and Table A3 respectively.

To evaluate the human activity recognition algorithm, we have calculated recall, precision, and F1-score of the model. As shown in Table 4, we have calculated the macro and micro averages of the model. Generally, when the dataset is imbalanced micro-average is used to evaluate the model. We have described more about these performance metrics in Appendix B. Table 5 depicts that the activity states, “Person entered”, “Person went away” and “Resting” have a greater number of false positives. The state “Person entered” is misclassified when the object detection module detects other objects such as couch as a person whereas “Person went away” is misclassified when the object detection module fails to detect a person. For example, if a person is standing in front of another person then the module fails to detect a person and the algorithm misclassifies the activity as “person went away”. Furthermore, when a person is sleeping on the couch and the camera is in such a position that it can capture only the head of the person then the algorithm misclassifies the activity as resting. However, the algorithm performs well in detecting true positive and true negative and has the overall accuracy of 93.87%. Furthermore, the computational time for human activity recognition given an input of image/frame sequences is around 2 s and for one image/frame the time is around 0.005 milliseconds.

Reference [56] provides a list of state-of-the-art methods for hand-crafted algorithms in the field of human action recognition. However, as discussed in the literature review section, references [33,34,35,36,37,38,39,40] use existing datasets, namely the Weizmann, Hollywood2, HMDB51, Olympic Sports, and UCF50, to validate their performance, and the main objective of these papers was to improve the performance of human action recognition. Our paper proposed to combine human activity and heat activity recognition with 3D thermal model reconstruction for smart home environments, and we proposed to use our own dataset. Thus, we decided to not compare our results with other methods.

We have applied Algorithm 5 to find the activity duration. The algorithm was applied to the information extracted for human and heat activity detection. The duration of each activity is shown in Table 6 and Table 7 for stove and human activity, respectively. As shown in the second row in Table 6 describes that the duration of the heating phase is 7 s, and the maximum temperature is 3 s. Similarly, the third row in Table 7, one person is resting for 111 s. This type of data can find many applications in HA systems. For instance, monitoring stove temperatures and their duration’s can help avoid hazardous situations in case they are accidentally left on (heating state), by automatically switching off the stove. Similarly, monitoring human activity duration and presence in a room can be integrated with HA systems to automatically switch off lighting, televisions, air conditioning systems, and HVAC when no one is present in the room, etc. hence saving power and energy.

**Figure 14:**
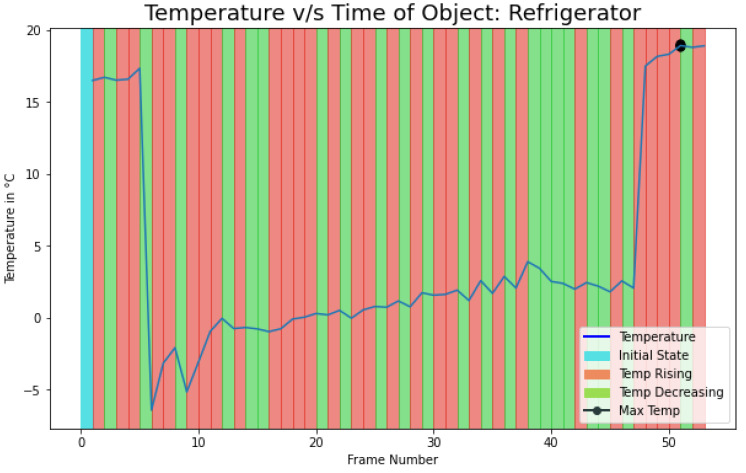
Refrigerator temperature fluctuations.

Furthermore, we conducted an experiment for heat activity recognition in the refrigerator. The idea was to detect the temperature fluctuation when the door is open and to find the duration of each heat activity. If the temperature in the refrigerator is increasing due to the high heat transfer rate, for more than a certain amount of duration, the user can be alerted that the refrigerator door should be closed. This result can be applied to save electricity and to improve the life of the compressor. As it can be seen from Figure 14 the temperature starts to increase with some fluctuation when the door is open (after frame number 5). The result of the activity duration on this experiment is shown in Table 8. Here, it can be depicted that as someone opens the door, there is a fluctuation in the temperature and the duration of the state temperature rising is more than temperature decreasing. More results for activity state and duration are attached in the Appendix A. (See Table A5 and Table A8.)

**Figure 15:**
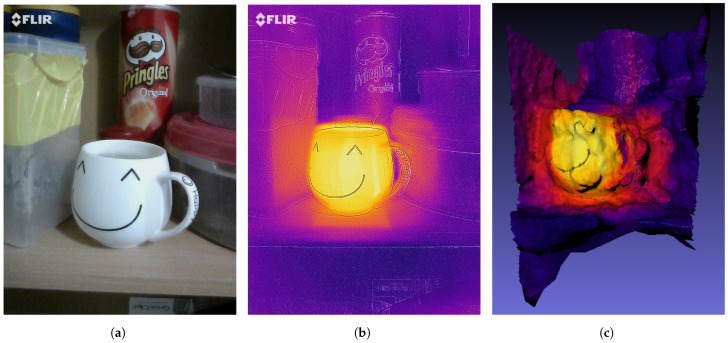
(**a**) Visual image, (**b**) Thermal image, and (**c**) 3D thermal model of a cupboard shelf.

A scene with a cup of hot water on the shelf of a cupboard was prepared as an experimental setup to validate Algorithms 6 and 7. A total of 15 stereo RGB images of the prepared scene was captured to generate the 3D thermal model of the scene. To capture stereo images, we have captured images from left and the right angle of a scene. These images were given as an input to Algorithm 6 that generated the 3D mesh of the scene. This 3D mesh and a thermal image were then given as an input to the Metashape to generate the 3D thermal model. The resulting 3D thermal model is shown in Figure 15c. One of the RGB images is shown in Figure 15a and the thermal image is shown in Figure 15b.

As described in the above section that the first and most important step of the 3D reconstruction is to find intrinsic and extrinsic camera parameters. The focal length is one of the important intrinsic camera parameters. The tool calculated the focal length of the RGB camera as 14.29 cm and the real value of the focal length for FLIR ONE PRO RGB camera is 15 cm [57]. Thus, the error is 0.71 cm. Due to this error, the depth map generated in the next step is not accurate resulting in some invalid 3D points in a dense cloud. To measure the quality of the 3D dense cloud, Root Mean Square Error of projection was used as a performance metric. The error calculated was 1.70 pixels. This shows that the generated 3D dense cloud was not accurate. The total number of pixels generated in a 3D dense cloud was 1,265,231. After getting a 3D dense cloud a 3D mesh was generated. As the 3D point cloud is not accurate, the 3D mesh generated using these points cannot generate a smooth surface which can be seen from Figure 15c. However, this phase has generated a 3D temperature distribution similar to the thermal image provided. For example, warm regions around the cup can be seen in both thermal image and 3D thermal model.

Using the generated 3D thermal model and the thermal image, as an input to Algorithm 7, we have extracted the temperature values for each coordinate. The overall process of temperature extraction takes more computational time as many points have new hue and cosine similarity takes time to find similar hue as it needs to compare current hue with all the other extracted hue from the image. Some example coordinates from the output are shown in Table 9. The coordinate system of the 3D mesh has X-axis as horizontal, Y-axis as vertical and Z-axis as depth axis. The origin (0,0,0) of the coordinate system of the cupboard shelf is located at the back wall of the cupboard on which the mug is placed and centered behind the mug. The 3D coordinates are shown in Table 9 which contains temperature in °C. The first three rows show temperature and coordinates of the heated mug, fourth row shows temperature around the mug whereas the last coordinate shows the temperature of the surrounding object which is at the room temperature.

**Figure 16:**
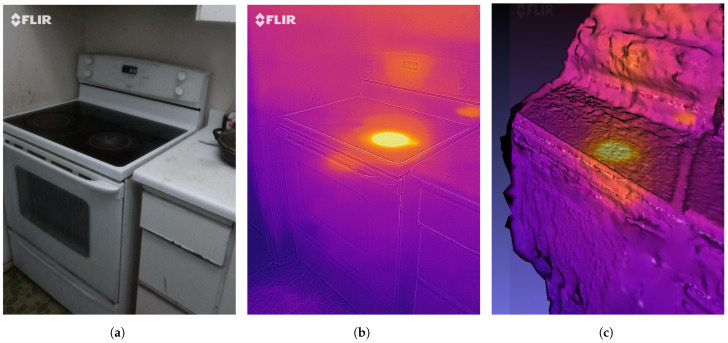
(**a**) Visual image, (**b**) Thermal image, and (**c**) 3D thermal model of a stove.

We have conducted another experiment for 3D thermal model generation. For this experiment, we have captured stereo images of a heated stove in the kitchen. A total of 8 stereo RGB images of the prepared scene was captured to generate the 3D thermal model of the scene. Using these images as an input to Algorithm 6 and Meshlab, a 3D thermal model was generated which is shown in Figure 16c. One of the RGB images is shown in Figure 16a and the thermal image is shown in Figure 16b. The number of pixels generated for the 3D dense point cloud was 5,825,320. The re-projection error calculated was 1.58 pixels. Due to this error, the resulting surface of the 3D mesh was not smooth as it can be depicted from Figure 16c. However, the heated region in the stove and around the digital clock above the stove can be seen in the 3D reconstructed thermal model. Moreover, the 3D coordinates with the corresponding temperature is shown in Table 10. The origin (0,0,0) of the coordinate system of the kitchen was located under the stove and behind the oven that is the reason for negative Z and X coordinates and positive Y coordinates for the heated stove. The first three rows show coordinates and temperature of the heated region of the stove, the fourth row depicts the temperature and coordinates of the warm region around the stove whereas the last row shows temperatures for the surrounding objects that are at room temperature. Data obtained from such a 3D thermal model can be very useful to determine the temperature in 3D space. This data can further be integrated with HA systems for energy management and risk prevention purposes. One can effectively detect any heat leakage and counter measures can be taken to save energy and to prevent dangerous situation.

We have conducted long duration experiments on a living space including a living room, a kitchen, and a cupboard to validate our algorithms. However, for the sake of brevity, we have presented only a very small portion of the results in Table 2, Table 3, Table 4, Table 5, Table 6, Table 7, Table 8, Table 9 and Table 10. For human activity recognition, we recorded a video of a living room for more than 30 min and extracted a total of 1495 video annotations from it, and recognized a total of 234 human activity states. Similarly, for stove activity recognition, we recorded a video for 15 min and extracted a total of 122 annotations from it, and recognized a total of 42 heat activity states from this video, and for heat activity recognition in refrigerator we recorded a video for 5 min and extracted a total of 156 annotations from it, and recognized a total of 53 heat activity states from this video. Furthermore, we reconstructed the 3D model with a total of 22,210 and 86,578 3D coordinates for the mug and kitchen experiment, respectively, and extracted the temperature of each of the coordinates as well. We have added some more results in Appendix A.

## 5. Challenges

The whole process of annotating the video/image with an object’s temperature value depends on the accuracy of object detection framework. Here, we have used tensorflow’s object detection APIs. The model is trained on general-purpose 1000 objects, such as ovens, tables, persons, and cars. For this paper, our main focus is to identify activities in a closed environment. Hence, there is a high probability that some of the objects present in the image will not be correctly classified. We assume that for the full implementation, the object detection module will be trained for the task-specific dataset. We also proposed to implement learning-based methods in the future when we gather the dataset for indoor environments.

Moreover, the following issues can be improved to provide a more descriptive state of human activity. (1) If a person is sleeping on the couch and the camera detects only some of the parts such that a square bounding box is created then the algorithm detects it as a resting activity rather than sleeping. This can be solved by placing two cameras in the top-left and top-right corner of a room and we can combine the results from both cameras such that the bounding box with high-confidence should be counted. (2) Missed-classification: If we had taken images at 1 or 5 frame/sec and then a person was missed-classified in one of the frames, then we can easily ignore it as it is unlikely that person can make significant movement within a small timeframe inside a home environment. Though in our case it could be possible that a person has moved so handling miss-classification becomes tougher. Therefore, ideally, the object detection module should have near human-level accuracy. (3) Tracking people: If two consecutive frames have 2 people in it then it becomes tougher to evaluate if a person has moved or not by simply looking at their locations in 2 frames as it might be possible that persons have swapped their locations.

The second module of this paper deals with the problem of 3D thermal model generation. In this module, to project the thermal image onto the 3D mesh, the thermal camera’s parameters are required. However, a thermal camera cannot be calibrated using the classical checkerboard method proposed in [13] as it requires detecting edges and corners in a chessboard and thermal images do not contain these kinds of features. Thus, to create a 3D thermal model, we have used a tool named Meshlab which requires human intervention to align the thermal image and 3D mesh such that their coordinates align, and then the image is projected onto the 3D mesh. To remove human intervention for 3D thermal model generation, the thermal camera calibration process can be done. Reference [8] proposed a method to calibrate the thermal camera using the specifically created thermal calibration rig. The calibration rig has LEDs located on each corner of the checkerboard and the heat of these LEDs can be detected in a thermal image. As this method allows the thermal image to detect corners of the checkerboard, thermal camera parameters can be calculated [8]. This process can reduce the error introduced during manual alignment by providing a way to automate the process resulting in more accurate results for the 3D thermal model.

Apart from this, the main requirement for the 3D mesh is that the input images should be stereo. The stereo images provide details of a scene from different angles. This information is used to extract the depth information of the scene which is then used to create depth maps. Using the stereo images, highly accurate depth maps and the dense cloud can be generated. If the camera is in a steady position then the proposed method produces erroneous results as it generates depth maps with minimal depth information. This problem can be resolved by mounting two cameras into two elevated corners of a room in a house or office such that images from the left and right of a scene can be captured.

## 6. Conclusions

This paper presents various algorithms for thermal and optical imagery-based activity recognition and 3D thermal model generation in an indoor environment. Activity recognition in indoor environments consists of two main problems: human activity recognition and household activity recognition. We have used both RGB and thermal images of a scene as RGB images provide features for object detection and thermal images provide information about the temperature. Thermal-RGB image pairs and stereo RGB images were captured using a FLIR ONE PRO camera for a scene and used to recognize human and household activity in residential spaces, to determine its duration, and to create a 3D thermal model of the environment. This was done by first detecting objects in a scene and then using this information in conjunction with thermal images to finally detect human and household activities. The human activity recognition’s micro recall, precision, and f1-score were measured as 95.24%, 78.08%, and 0.86, respectively. In addition to that, the accuracy of the algorithm was measured as 93.87%. An algorithm was also designed to find activity duration which can be used by the alert systems to improve safety. The proposed algorithms were validated by conducting various experiments in an IoT-based setup. A 3D thermal model was generated to get precise 3D heat distribution of an environment as a 2D thermal image provides limited information. This was done in three steps: (a) 3D dense cloud reconstruction, (b) 3D mesh generation, and (c) 3D thermal model generation. These steps were performed using the tool Metashape by Agisoft and Meshlab. Generated 3D thermal models were accurate with re-projection root mean square error as small as 1.58 pixels. The accuracy of the 3D thermal model depends on the accuracy of the 3D dense cloud and 3D mesh.

The algorithms and experiments on activity recognition and 3D thermal model generation presented in this paper can be readily used in home automation, healthcare, monitoring, security, and surveillance systems. The proposed algorithms detect human and heat activities which can be used to make a safe smart home. Results of both algorithms can be combined to prevent any hazardous situation. For an instance, if a person is sleeping and a heat leakage is detected then the person can be alerted using other alert systems to prevent any accident or if possible the system can turn off the hazardous electrical appliances automatically to prevent further damage.

This work can further be enhanced by creating a dataset and training a task-specific object detection model to solve the problem of undetected objects due to closed environments. Furthermore, the proposed algorithm for activity recognition is a non-learning-based approach due to a lack of dataset. In future work, we proposed to create a dataset and use machine learning-based methods to detect activity in the household environment. Moreover, our approach for 3D thermal model construction requires the manual projection of the thermal image onto the 3D mesh. This can be automated by finding parameters of the thermal camera so that no human intervention is required for building a 3D model. These parameters can also improve the accuracy of the 3D thermal model.

## Figures and Tables

**Table 1:** Different techniques used in different papers for human and household activity recognition.

Techniques	Human Activity Recognition	Household Activity Recognition
Using Thermal Images	Standing [21], Sitting [24], Walking [22,23]	Stove Activity [5], Pet Detection [26]
Using RGB Images	Lying Down [20]	**-**
Using Sensor Data	Sitting [32], Standing [18,25]	Stove Activity [19]
Using Deep Learning	Standing [21]	**-**
Using Machine Learning	Lying Down [20], Standing [18,25], Sitting [24,32], Walking [22,23]	Stove Activity [19]
Using Custom Algorithms	**-**	Stove Activity [5], Pet Detection [26]
Determining Activity Duration	Sitting [32]	Stove Activity [5,19]

**Table 2:** Stove Activity states for sample frames.

Frame No.	Activity State
1	Initial state and no activity
3	Heating phase (Temperature Increasing)
12	Maximum Temperature
18	Cooling phase (Temperature Decreasing)

**Table 3:** Human Activity States for sample frames.

Frame No.	Activity State
2	No person in the frame
3	1 person entered. 0 sleeping. 1 standing. 0 resting. Total person:1
4	0 sleeping. 0 standing. 1 resting. Total person:1
26	1 more person entered. Total person:2. 0 sleeping. 0 standing. 2 resting. Total person:2
59	0 sleeping. 1 standing. 1 resting. Total person:2
60	1 person went away. Total person:1. 0 sleeping. 0 standing. 1 resting. Total person:1
148	1 sleeping. 0 standing. 0 resting. Total person:1
153	0 sleeping. 1 standing. 0 resting. Total person:1
232	1 person went away. Total person: 0. No person in the frame.
233	No person in the frame

**Table 4:** Performance metrics for human activity recognition.

Average-Type	Recall (%)	Precision (%)	F1-Score	Accuracy (%)
Macro	88.74	73.05	0.80	93.87
Micro	**95.24**	**78.08**	**0.86**	

**Table 5:** Results for human activity recognition.

Activity State	True Positive	False Positive	True Negative	Negative Falses
	(TP)	(FP)	(TN)	(FN)
No person in the frame	59	1	174	0
Person entered	10	27	195	2
Person went away	10	29	191	4
Sleeping	10	0	223	1
Standing	35	1	193	5
Resting	136	15	82	1

**Table 6:** Stove activity duration for sample frames.

Activity State	Activity Duration (s)
Initial state and no activity	0
Heating phase (Temperature Increasing)	7
Maximum Temperature	3
Cooling phase (Temperature Decreasing)	1

**Table 7:** Human activity duration for sample frames.

Activity State	Activity Duration (s)
No person in the frame	10
1 person entered. 0 sleeping. 1 standing. 0 resting. Total person:1	5
0 sleeping. 0 standing. 1 resting. Total person:1	111
1 more person entered. Total person:2. 0 sleeping. 0 standing. 2 resting. Total person:2	5
1 person went away. Total person:1. 0 sleeping. 0 standing. 1 resting. Total person:1	5
0 sleeping. 0 standing. 1 resting. Total person:1	30
1 person went away. Total person: 0	5
1 person went away. Total person: 0. No person in the frame.	10

**Table 8:** Refrigerator activity duration for sample frames.

Activity State	Activity Duration (s)
Initial State	5
Temp Rising	6
Temp Decreasing	5
Temp Rising	9
Temp Decreasing	5
Temp Rising	11

**Table 9:** Temperature of 3D world coordinates of the cupboard shelf.

X	Y	Z	Temperature (°C)
0.62	−1.51	−6.13	36.05
0.60	−1.50	−6.13	35.38
0.64	−1.43	−6.12	34.56
1.65	−0.98	−6.30	26.17
−1.59	−2.66	−6.56	13.51

**Table 10:** Temperature of 3D world coordinates of the kitchen.

X	Y	Z	Temperature (°C)
−2.62	4.82	−5.35	74.43
−1.28	6.63	−3.39	53.29
−1.64	5.24	−3.98	47.64
−1.24	6.40	−3.33	39.28
−0.38	4.02	−3.73	22.02

## Data Availability

Not applicable.

## Abbreviations

The following abbreviations are used in this manuscript:

HAR	Human Activity Recognition
IoT	Internet of Things
HA	Human Automation
ML	Machine Learning
GEI	Gain Energy Image
PCA	Principal Component Analysis
MDA	Multiple Discriminant Analysis
CNN	Convolutional Neural Network
RNN	Recurrent Neural Network
SFM	Structure From Motion
SIFT	Scale-Invariant Feature Transform
RANSAC	Random Sample Consensus
MSAC	M-estimator Sample Consensus
USB	Universal Serial Bus
API	Application Programming Interface
CSV	Comma Separated Values

## Appendix A. Supporting Results

**Table A1:** Video annotations of the living room (40 out of 1495 examples).

Object	X	Y	Width	Height	Timestamp	Max Temp	Min Temp	Mean Temp	Frame
bottle	481.82	246.68	19.93	45.1	16:37:41	21.99	17.39	20.15	1
cup	418.97	258.41	15.96	19.74	16:37:41	21.95	21.03	21.39	1
tv	207.67	102.25	127.61	115.95	16:37:41	39.7	19.85	22.65	1
tv	289.37	103.94	53.32	101.52	16:37:41	22.7	19.95	21.84	1
bottle	485.76	245.31	18.28	45.09	16:37:46	21.92	17.29	20.28	2
tv	208.45	98.55	135.81	117.01	16:37:46	38.84	20.03	22.55	2
cup	421.32	257.32	18.44	20.06	16:37:46	22.09	20.74	21.32	2
person	132.64	28.2	168.42	425.67	16:37:51	31.59	12.97	24.03	3
couch	273.47	193.35	280.71	283.87	16:37:51	31.29	12.97	22.5	3
cup	113.6	281.27	15.87	20.32	16:37:51	22.02	21.3	21.66	3
bottle	132.03	272.27	12.93	34.08	16:37:51	21.88	18.53	21.16	3
person	188.76	165.8	246.37	237.6	16:37:56	33.78	12.47	22.25	4
couch	295.99	203.51	288.52	272.83	16:37:56	33.06	17.99	22.79	4
tv	0.82	122.36	58.91	112.61	16:37:56	22.3	20.35	21.82	4
cup	138.29	282.16	17.49	21.49	16:37:56	22.02	20.93	21.5	4
bottle	199.76	267.12	18.48	44.07	16:37:56	22.09	17.41	20.49	4
cup	158.05	284.28	11.47	23.87	16:37:56	21.84	20.78	21.5	4
person	185.58	165.03	226.64	228.75	16:38:01	34.32	12.07	22.6	5
tv	1.62	118.45	58.78	111.81	16:38:01	22.31	20.14	21.81	5
couch	300.4	201.48	285.36	275.88	16:38:01	33.55	16.21	22.99	5
bottle	200.06	264.6	18.29	43.75	16:38:01	22.39	17.35	20.5	5
cup	139.09	278.02	16.62	21.11	16:38:01	22.06	20.85	21.36	5
cat	285.13	242.21	52.19	39.24	16:38:01	32.1	18.36	23.17	5
person	300.13	150.17	237.7	241.04	16:38:06	33.58	13.04	22.52	6
couch	420.73	202.19	218.28	276.41	16:38:06	31.99	18.0	22.98	6
tv	32.73	115.52	147.96	114.19	16:38:06	37.97	19.74	22.56	6
bottle	315.46	258.57	15.97	44.16	16:38:06	22.57	17.68	20.4	6
cup	254.61	272.39	16.85	19.84	16:38:06	21.94	21.09	21.43	6
person	297.75	162.16	239.3	231.5	16:38:11	34.01	14.2	22.76	7
cup	322.92	270.64	63.15	63.26	16:38:11	23.73	14.87	22.08	7
couch	419.74	211.35	220.26	268.65	16:38:11	32.26	17.92	23.18	7
tv	33.31	119.79	141.71	116.65	16:38:11	37.3	19.89	22.73	7
bottle	313.27	268.28	15.97	43.48	16:38:11	22.39	17.74	20.88	7
cup	252.62	281.82	16.47	19.34	16:38:11	21.9	21.1	21.45	7
dining table	190.83	284.37	151.67	37.29	16:38:11	22.79	16.86	21.49	7
person	293.94	146.54	239.23	241.74	16:38:16	33.32	13.04	22.27	8
bottle	311.21	253.83	19.1	42.24	16:38:16	22.36	17.58	20.44	8
couch	414.75	197.81	224.2	275.75	16:38:16	31.69	18.05	22.81	8
tv	32.44	112.3	142.35	118.14	16:38:16	37.42	20.11	22.53	8
cup	252.16	268.36	16.09	19.24	16:38:16	21.82	20.75	21.26	8

**Table A2:** Video annotations of the kitchen (40 out of 122 examples).

Object	X	Y	Width	Height	Timestamp	Max Temp	Min Temp	Mean Temp	Frame
oven	3.32	23.02	470.55	604.51	15:53:13	25.72	23.62	24.62	1
oven	12.94	14.01	416.66	245.26	15:53:13	25.72	24.2	24.71	1
oven	2.12	7.57	410.87	117.05	15:53:13	24.96	24.11	24.62	1
microwave	3.21	6.05	415.41	127.18	15:53:13	24.96	24.11	24.62	1
oven	3.04	27.35	472.57	612.65	15:53:13	25.62	23.73	24.66	2
oven	17.36	12.25	398.45	123.22	15:53:13	25.04	24.15	24.63	2
oven	7.78	85.31	472.22	533.6	15:53:14	46.44	23.96	25.52	3
oven	8.87	302.24	453.28	325.84	15:53:14	46.44	23.94	26.01	3
microwave	36.73	99.96	387.34	140.47	15:53:14	25.37	24.24	24.92	3
oven	3.12	28.52	468.01	597.4	15:53:14	130.11	26.61	30.57	5
microwave	9.49	38.62	385.28	147.89	15:53:14	29.6	26.86	27.17	5
oven	10.92	11.11	452.56	621.36	15:53:14	160.04	26.92	32.02	6
microwave	8.22	41.12	385.88	146.15	15:53:14	30.29	27.22	27.54	6
oven	2.52	42.76	474.53	597.24	15:53:16	182.63	27.12	33.2	7
microwave	10.29	39.66	388.31	154.56	15:53:16	32.52	27.46	27.81	7
oven	4.92	35.37	469.45	604.63	15:53:21	226.21	26.64	35.2	8
microwave	19.49	34.63	377.81	152.34	15:53:21	36.17	27.14	27.94	8
oven	7.02	42.04	458.08	591.47	15:53:22	226.21	26.69	35.48	9
microwave	15.61	47.26	377.38	134.25	15:53:22	32.43	27.16	27.92	9
oven	8.0	43.41	453.44	582.03	15:53:23	226.21	26.63	35.55	10
microwave	9.66	43.51	376.93	139.79	15:53:23	33.11	27.08	27.81	10
oven	5.37	494.2	436.45	139.0	15:53:23	28.47	26.62	27.16	10
oven	9.71	12.47	459.83	614.76	15:53:24	226.21	26.37	34.71	11
oven	16.54	18.39	395.72	120.28	15:53:24	30.38	27.03	27.75	11
oven	7.51	9.28	457.57	630.72	15:53:24	226.21	26.3	34.39	12
oven	3.97	28.26	468.81	602.83	15:53:24	194.45	25.1	32.38	13
microwave	0.0	1.33	406.02	114.39	15:53:24	27.98	25.89	26.76	13
oven	5.28	22.44	474.01	606.97	15:53:24	193.33	25.0	32.12	14
oven	12.3	8.42	410.51	232.79	15:53:24	31.17	25.82	26.75	14
microwave	10.33	6.05	408.84	143.89	15:53:24	31.17	25.84	26.71	14
oven	11.37	34.72	456.68	592.91	15:53:24	180.45	25.15	32.02	15
microwave	19.74	36.61	381.16	148.04	15:53:24	30.98	25.72	26.8	15
oven	25.69	27.59	388.27	182.22	15:53:24	31.22	25.71	26.79	15
oven	4.51	57.21	461.19	577.79	15:53:24	173.35	25.34	32.04	16
oven	8.68	35.48	384.78	151.16	15:53:24	30.25	25.7	26.82	16
oven	9.01	33.05	453.85	590.95	15:53:25	168.78	25.2	31.51	17
microwave	25.25	36.87	380.1	152.9	15:53:25	29.35	25.55	26.67	17
oven	7.69	17.29	472.31	620.37	15:53:25	161.46	25.13	30.75	18
microwave	13.67	26.72	400.06	132.58	15:53:25	27.57	25.54	26.6	18
oven	19.15	16.09	399.29	160.65	15:53:25	29.86	25.52	26.6	18

**Table A3:** Human Activity States for sample frames (40 out of 234 examples).

Frame No.	Activity State
1	No person in the frame
2	No person in the frame
3	1 person entered. 0 sleeping. 1 standing. 0 resting. Total person:1
4	0 sleeping. 0 standing. 1 resting. Total person:1
5	0 sleeping. 0 standing. 1 resting. Total person:1
6	0 sleeping. 0 standing. 1 resting. Total person:1
7	0 sleeping. 0 standing. 1 resting. Total person:1
8	0 sleeping. 0 standing. 1 resting. Total person:1
9	0 sleeping. 0 standing. 1 resting. Total person:1
10	0 sleeping. 0 standing. 1 resting. Total person:1
11	0 sleeping. 0 standing. 1 resting. Total person:1
12	0 sleeping. 0 standing. 1 resting. Total person:1
13	0 sleeping. 0 standing. 1 resting. Total person:1
14	0 sleeping. 0 standing. 1 resting. Total person:1
15	0 sleeping. 0 standing. 1 resting. Total person:1
16	0 sleeping. 0 standing. 1 resting. Total person:1
17	0 sleeping. 0 standing. 1 resting. Total person:1
18	0 sleeping. 0 standing. 1 resting. Total person:1
19	0 sleeping. 0 standing. 1 resting. Total person:1
20	0 sleeping. 0 standing. 1 resting. Total person:1
21	0 sleeping. 0 standing. 1 resting. Total person:1
22	0 sleeping. 0 standing. 1 resting. Total person:1
23	0 sleeping. 0 standing. 1 resting. Total person:1
24	0 sleeping. 0 standing. 1 resting. Total person:1
25	0 sleeping. 0 standing. 1 resting. Total person:1
26	1 more person entered. Total person:2. 0 sleeping. 0 standing. 2 resting. Total person:2
27	1 person went away. Total person:1. 0 sleeping. 0 standing. 1 resting. Total person:1
28	0 sleeping. 0 standing. 1 resting. Total person:1
29	0 sleeping. 0 standing. 1 resting. Total person:1
30	0 sleeping. 0 standing. 1 resting. Total person:1
31	0 sleeping. 0 standing. 1 resting. Total person:1
32	0 sleeping. 0 standing. 1 resting. Total person:1
33	0 sleeping. 0 standing. 1 resting. Total person:1
34	1 person went away. Total person: 0. No person in the frame.
35	No person in the frame
36	No person in the frame
37	1 person entered. 0 sleeping. 1 standing. 0 resting. Total person:1
38	0 sleeping. 0 standing. 1 resting. Total person:1
39	1 more person entered. Total person:2. 0 sleeping. 1 standing. 1 resting. Total person:2
40	0 sleeping. 0 standing. 2 resting. Total person:2

**Table A4:** Stove States for sample frames (42 out of 42 examples).

Frame No.	Activity State
3	Initial
3	Initial
5	Temp Rising
6	Temp Rising
7	Temp Rising
8	Max Temp
9	Max Temp
10	Max Temp
11	Max Temp
12	Max Temp
13	Temp Decreasing
14	Temp Decreasing
15	Temp Decreasing
16	Temp Decreasing
17	Temp Decreasing
18	Temp Decreasing
19	Temp Decreasing
20	Temp Decreasing
21	Temp Decreasing
22	Temp Decreasing
22	Temp Decreasing
23	Temp Rising
24	Temp Decreasing
25	Temp Decreasing
25	steady
26	Temp Decreasing
26	Temp Decreasing
27	Temp Rising
28	Temp Decreasing
29	Temp Decreasing
29	Temp Decreasing
30	Temp Decreasing
30	steady
31	Temp Rising
32	Temp Decreasing
33	Temp Decreasing
33	steady
34	Temp Decreasing
35	Temp Decreasing
35	Temp Decreasing
36	Temp Rising
37	Temp Decreasing

**Table A5:** Refrigerator States for sample frames (53 out of 53 examples).

Frame No.	Activity State
1	Initial
2	Temp Rising
3	Temp Decreasing
4	Temp Rising
5	Temp Rising
6	Temp Decreasing
7	Temp Rising
8	Temp Rising
9	Temp Decreasing
10	Temp Rising
11	Temp Rising
12	Temp Rising
13	Temp Decreasing
14	Temp Rising
15	Temp Decreasing
16	Temp Decreasing
17	Temp Rising
18	Temp Rising
19	Temp Rising
20	Temp Rising
21	Temp Decreasing
22	Temp Rising
23	Temp Decreasing
24	Temp Rising
25	Temp Rising
26	Temp Decreasing
27	Temp Rising
28	Temp Decreasing
29	Temp Rising
30	Temp Decreasing
31	Temp Rising
32	Temp Rising
33	Temp Decreasing
34	Temp Rising
35	Temp Decreasing
36	Temp Rising
37	Temp Decreasing
38	Temp Rising
39	Temp Decreasing
40	Temp Decreasing
41	Temp Decreasing
42	Temp Decreasing
43	Temp Rising
44	Temp Decreasing
45	Temp Decreasing
46	Temp Rising
47	Temp Decreasing
48	Temp Rising
49	Temp Rising
50	Temp Rising
51	Max Temp
52	Temp Decreasing
53	Temp Rising

**Table A6:** Human Activity Duration for sample frames (40 out of 142 examples).

Activity State	Activity Duration (s)
No person in the frame	10
1 person entered. 0 sleeping. 1 standing. 0 resting. Total person:1	5
0 sleeping. 0 standing. 1 resting. Total person:1	111
1 more person entered. Total person:2. 0 sleeping. 0 standing. 2 resting. Total person:2	5
1 person went away. Total person:1. 0 sleeping. 0 standing. 1 resting. Total person:1	5
0 sleeping. 0 standing. 1 resting. Total person:1	5
1 person went away. Total person: 0. No person in the frame.	5
No person in the frame	10
1 person entered. 0 sleeping. 1 standing. 0 resting. Total person:1	5
0 sleeping. 0 standing. 1 resting. Total person:1	5
1 more person entered. Total person:2. 0 sleeping. 1 standing. 1 resting. Total person:2	5
0 sleeping. 0 standing. 2 resting. Total person:2	11
1 more person entered. Total person:3. 0 sleeping. 0 standing. 3 resting. Total person:3	4
1 person went away. Total person:2. 0 sleeping. 0 standing. 2 resting. Total person:2	6
1 more person entered. Total person:3. 0 sleeping. 0 standing. 3 resting. Total person:3	5
1 person went away. Total person:2. 0 sleeping. 0 standing. 2 resting. Total person:2	4
0 sleeping. 0 standing. 2 resting. Total person:2	11
1 person went away. Total person:1. 0 sleeping. 1 standing. 0 resting. Total person:1	5
2 more person entered. Total person:3. 0 sleeping. 0 standing. 3 resting. Total person:3	5
0 sleeping. 0 standing. 3 resting. Total person:3	5
1 person went away. Total person:2. 0 sleeping. 0 standing. 2 resting. Total person:2	5
0 sleeping. 1 standing. 1 resting. Total person:2	4
1 more person entered. Total person:3. 0 sleeping. 2 standing. 1 resting. Total person:3	6
0 sleeping. 1 standing. 2 resting. Total person:3	4
1 more person entered. Total person:4. 0 sleeping. 2 standing. 2 resting. Total person:4	5
0 sleeping. 0 standing. 4 resting. Total person:4	6
1 person went away. Total person:3. 0 sleeping. 0 standing. 3 resting. Total person:3	4
1 person went away. Total person:2. 0 sleeping. 0 standing. 2 resting. Total person:2	6
0 sleeping. 1 standing. 1 resting. Total person:2	5
1 person went away. Total person:1. 0 sleeping. 0 standing. 1 resting. Total person:1	5
1 more person entered. Total person:2. 0 sleeping. 1 standing. 1 resting. Total person:2	5
0 sleeping. 1 standing. 1 resting. Total person:2	4
0 sleeping. 0 standing. 2 resting. Total person:2	5
1 more person entered. Total person:3. 0 sleeping. 0 standing. 3 resting. Total person:3	5
1 person went away. Total person:2. 0 sleeping. 0 standing. 2 resting. Total person:2	5
0 sleeping. 0 standing. 2 resting. Total person:2	11
1 more person entered. Total person:3. 0 sleeping. 0 standing. 3 resting. Total person:3	4
1 more person entered. Total person:4. 0 sleeping. 0 standing. 4 resting. Total person:4	5
0 sleeping. 0 standing. 4 resting. Total person:4	11
1 person went away. Total person:3. 0 sleeping. 0 standing. 3 resting. Total person:3	5
0 sleeping. 0 standing. 3 resting. Total person:3	6

**Table A7:** Stove activity duration for sample frames (16 out of 16 examples).

Activity State	Activity Duration (s)
Initial	0
Temp Rising	7
Max Temp	3
Temp Decreasing	1
Temp Rising	0
Temp Decreasing	1
steady	0
Temp Decreasing	0
Temp Rising	0
Temp Decreasing	0
steady	1
Temp Rising	1
Temp Decreasing	1
steady	1
Temp Decreasing	1
Temp Rising	0

**Table A8:** Refrigerator activity duration for sample frames (35 out of 35 examples).

Activity State	Activity Duration (s)
Initial	5
Temp Rising	6
Temp Decreasing	5
Temp Rising	9
Temp Decreasing	5
Temp Rising	11
Temp Decreasing	4
Temp Rising	16
Temp Decreasing	5
Temp Rising	5
Temp Decreasing	10
Temp Rising	20
Temp Decreasing	5
Temp Rising	5
Temp Decreasing	5
Temp Rising	10
Temp Decreasing	5
Temp Rising	5
Temp Decreasing	4
Temp Rising	5
Temp Decreasing	6
Temp Rising	10
Temp Decreasing	5
Temp Rising	5
Temp Decreasing	5
Temp Rising	5
Temp Decreasing	5
Temp Rising	5
Temp Decreasing	20
Temp Rising	5
Temp Decreasing	10
Temp Rising	5
Temp Decreasing	5
Temp Rising	15
Max Temp	5
Temp Decreasing	5
Temp Rising	5

**Table A9:** Temperature of 3D world coordinates of the cupboard (20 out of 22,210 examples).

X	Y	Z	Temperature (°C)
2.0014	0.6398	−7.9673	14.6988
0.4906	−1.928	−6.0505	34.7017
1.7723	−2.871	−6.2441	13.7303
−1.3437	−0.8388	−7.1432	27.2098
0.5947	−0.7864	−5.999	35.4228
0.4679	−0.7649	−5.7605	35.7059
0.0798	−0.5405	−5.8944	35.7748
0.9333	−1.7619	−6.3756	27.7674
0.4979	2.0293	−9.1158	13.9691
−1.7164	−4.2573	−7.0667	13.8242
1.4497	−0.7472	−5.7463	13.6705
0.7368	2.4214	−8.905	14.2158
2.5194	−2.0902	−7.1275	14.6988
−0.1296	2.4935	−9.5485	18.518
2.7819	−0.1195	−6.0593	13.8242
1.1998	−1.2806	−6.5414	31.9194
−0.0629	−0.0563	−7.4047	24.4913
1.5232	2.1706	−9.1267	14.6988
1.9448	1.9133	−9.5913	14.6988
−1.1405	1.0634	−6.7961	13.6363

**Table A10:** Temperature of 3D world coordinates of kitchen (20 out of 86,578 examples).

X	Y	Z	Temperature (°C)
−1.9661	0.11	−4.8897	35.4505
−2.6144	6.0175	−5.2324	49.4435
−2.2303	−0.4009	−5.2172	105.2417
−2.3037	7.0193	−4.7563	45.6503
−2.1104	−0.1235	−4.9684	35.4505
0.2324	8.4416	−2.1597	105.2417
−2.081	0.7635	−4.9218	62.018
−0.2367	2.4341	−4.2632	22.0286
−2.2353	4.7756	−4.8054	22.1461
−1.6952	6.5153	−3.9385	22.8406
−6.831	3.6134	−7.1942	22.0442
−3.7903	10.0444	−5.5572	22.0286
0.3989	11.0971	−1.8679	21.9658
−4.4265	10.4278	−5.1591	22.0286
−3.2999	2.6144	−6.417	22.2947
−4.7288	0.011	−8.5779	22.0286
−5.887	0.6152	−8.4636	105.3403
−3.886	1.8818	−7.2735	128.4772
−3.8856	1.1329	−7.3805	41.9647
−4.2032	9.2512	−5.5857	105.3403

## Appendix B. Performance Metrics

For all the performance metrics written below, True Positive (TP) is the number of observations that belong to a positive class and are correctly classified by the algorithm and True Negative (TN) is the number of observations that belong to a negative class and are correctly classified by the algorithm. False Positive (FP) is the number of observations that are misclassified to the positive class and False Negative (FN) is the number of observations that are misclassified to the negative class.

(A1) $$Accuracy=\frac{TP+TN}{TP+TN+FP+FN}$$

Here, the *Accuracy* is the ratio of the number of true predictions of the algorithm to the total number of observations.

(A2) $$Recall=\frac{TP}{TP+FN}$$

Here, the *recall* is the ratio of the number of true positive predicted by the algorithm to the total number of observations that actually belong to positive class.

(A3) $$Precision=\frac{TP}{TP+FP}$$

Here, *Precision* is the ratio of the number of true positive to the total number of observations classified as true.

(A4) $$F1-Score=2\cdot \frac{Precision\cdot Recall}{Precision+Recall}$$

Here, *F*1-*score* is the harmonic average of precision and recall.

When we deal with multi-class problems we generally average these performance metrics among all classes. For that, we have two types: one is macro-average and the other is micro-average. Macro-average is calculated by taking the average of the performance metric of individual classes. For instance, macro-recall can be calculated by taking the average of recall of each class. Macro-average gives the same weight to each of the classes during the average. When the dataset is imbalanced then we use micro-average. Let us say we have a k-class classification problem then the micro-average is calculated as shown in the below equations.

(A5) $$Micro\_Recall=\frac{\sum_{i=1}^{k}TP_{k}}{\sum_{i=1}^{k}TP_{k}+FN_{k}}$$

Here, micro recall is the ratio of the sum of the *TP* of all classes to the sum of the *TP* and the *FN* of all classes

(A6) $$Micro\_Precision=\frac{\sum_{i=1}^{k}TP_{k}}{\sum_{i=1}^{k}TP_{k}+FP_{k}}$$

Here, micro precision is the ratio of the sum of the *TP* of all classes to the sum of the *TP* and the *FP* of all classes

(A7) $$Micro\_F1-Score=2\cdot \frac{Micro\_Precision\cdot Micro\_Recall}{Micro\_Precision+Micro\_Recall}$$

Here, micro *F*1-*Score* is the harmonic average of the micro precision and micro recall.
